# Physical Properties of Flours Obtained from Wasted Bread Crusts and Crumbs

**DOI:** 10.3390/foods10020282

**Published:** 2021-01-31

**Authors:** Juan Fernández-Peláez, Priscila Guerra, Cristina Gallego, Manuel Gomez

**Affiliations:** Food Technology Area, College of Agricultural Engineering, University of Valladolid, 34004 Palencia, Spain; juan.fernandez.pelaez@uva.es (J.F.-P.); priscilaguerra92@gmail.com (P.G.); cristina.gallego@uva.es (C.G.)

**Keywords:** leftovers, hydration properties, gel properties, rheology, stale bread

## Abstract

One third of the food produced in the world is wasted. Bread is one of the most wasted foods both during the distribution process and in households. To use these breads, it is necessary to get to know the properties of the flours that can be obtained from them. The purpose of this work is to know how the type of bread and its zone (crumb or crust) influence the characteristics of the flours obtained from the wasted bread. For this, flours made from the crumbs and crusts of eight different breads have been analysed. Their hydration properties, cold and post-heating rheology and gelling properties as well as the colour of flours and gels have been studied. Bread flours present higher water-holding capacity (WHC) and water-binding capacity (WBC) values and higher elastic modulus (G’) and viscous modulus (G”) values, both in cold conditions and after heating, than wheat flours. However, they generate weaker gels. Crust flours, and the gels obtained from them, are darker than those from crumbs and their gels. In terms of hydration and rheology, pan and wholemeal bread flours are generally lower than other bread flours. These flours also generate softer gels, possibly caused by the dilution of starch with other components. It can be concluded that the properties shown by wasted bread flours allow them to be reintroduced in the food chain as an ingredient in different products.

## 1. Introduction

It is estimated that one third of the world’s food production is wasted. In Europe, this represents 88 Mt or almost 175 kg per person per year, which amounts to about EUR 143 billion [1]. Such losses imply a high environmental impact, which can be reduced by minimizing these wastes or through better practices in their handling [2]. It is known that meat and bread wastage in supermarkets shows the highest environmental footprint [3]. One of the most recent studies conducted in Sweden reported that bread waste was 8.1 kg per person/year and it was concentrated at household and retail levels [4].

The European Union advises that when it is not possible to minimise it, food waste should preferably be allocated to human consumption, either directly or after its conversion into new products [5]. However, research into bread waste exploitation is focused on its use as animal feed [6,7] and in anaerobic digestion [8] or to produce ethanol [9,10]. Studies on the use of bread waste in human consumption are scarce and limited to its incorporation into extruded snacks [11] and sourdough [12].

In the baking process, since flour is hydrated and heated and thus allows the denaturation of gluten, it loses baking aptitude and starch gelatinisation. These processes are different in breadcrumbs and bread crust due to the faster drying of this area and the higher temperatures reached in baking, contributing to Maillard and sugar caramelisation reactions [13,14]. Although after starch gelatinisation, flours lose their potential to produce certain goods, biscuits can be prepared with flours that have undergone hydrothermal treatments [15]. These flours also show advantages for making batter mixes, increasing the pick-up [16]. In the same way, it is possible to make sauces in cold preparations with them [17]. However, the characteristics of wheat flours subjected to hydrothermal treatments differ depending on the treatment conditions [18]. To establish the suitability of the flours for processing, both their particle size and their water absorption capacity, in the case of biscuits, are important [19], as well as their rheological characteristics, in the case of batter and sauces [17,20]. Moreover, it is important to know whether different types of breads generate flours with similar properties and, therefore, can be mixed to obtain flours with uniform characteristics or whether they must be separated to obtain different types of flour.

The aim of this work is to analyse the characteristics of flours from stale breads (SBs), taking into account the differences between several types of breads and also between the internal (crumb) and the external (crust) parts of the breads. Therefore, eight types of breads that differ in their ingredients, sizes of pieces and hydration of the dough have been selected, and the properties (particle size, colour, rheology, hydration, pasting and gel properties) of the flours obtained from their crumbs and crusts have been assessed. These properties were compared with those of a commercial wheat flour.

## 2. Materials and Methods

### 2.1. Materials

Bread and strong wheat flour were purchased at Carrefour hypermarket in Palencia (Spain). All bread was purchased twice, on different days, at least two weeks apart, to have a repetition. The main ingredients of the breads are shown in Table 1.

To perform the analyses, crumbs and crust were separated from the bread and left to dry on trays at room temperature for 24 h. They were later milled in an LM 3100 hammer mill (Perten Instruments, Huddinge, Sweden).

### 2.2. Methods

#### 2.2.1. Particle Size, Microstructure and Colour of the Flours

The particle size of the flours was evaluated using the particle size analyser Mastersizer 3000 (Malvern Instruments, Malvern, United Kingdom). The values of D [4,3] represent the equivalent spherical diameter of the particles. The measurements were taken in duplicate.

Photographs of the flour particles were taken with a scanning electron microscope (SEM), Quanta 200FEI (Hillsboro, Oregon, USA). Microphotographs were obtained in secondary electron detection mode at 3 KeV under high vacuum conditions with a backscattered electron detector (BSED).

The colour of the flours and gels was measured with the PCE-CSM 2 colorimeter (PCE Instruments, Meschede, Germany) employing the D65 Illuminant with a 2° Standard Observer. The parameters determined were L* (lightness), a* (green–red axis) and b* (blue–yellow axis). All flours and gels were measured in duplicate.

#### 2.2.2. Hydration Properties

Water-holding capacity (WHC) is defined as the amount of water retained by a sample in the absence of stress. Swelling volume (SV) is the increase in volume occupied by a given amount of sample after undergoing hydration. Both parameters were measured according to de la Hera et al. (2013) [21], with certain modifications. Briefly, 100 mL of distilled water was added to 5 g of the sample and left to hydrate for 24 h. At that point, the volume of the sample was measured, and the hydrated solid was weighed after removing the excess water. WHC was determined as the amount of water retained by the dry sample. SV was calculated as the volume per weight of dry sample.

Water-binding capacity (WBC) is the amount of water retained by a sample after it has been subjected to centrifugation. This parameter was measured in duplicate according to method 56-30.01 [22] with modifications. Briefly, 5 g of sample and 25 mL of distilled water were mixed. The mixture was centrifuged at 2000 rpm for 10 min and the supernatant was removed. WBC was calculated as the amount of water retained per dry sample.

Water absorption index (WAI), the grams of sediment per gram of sample, was determined following the method of Rosell et al. (2011) [23] with modifications. Briefly, 2.5 g (±0.1 g) of sample was dispersed in 30 mL of distilled water in Falcon tubes and heated to 90 °C for 15 min. After this, the hydrated and heated sample was left to cool for 1 h. Subsequently, the tubes were centrifuged at 3000 rpm for 10 min at 4 °C. Finally, the resulting supernatant was decanted and the residue from the Falcon tube was weighed. The weight of the dry solids was measured after evaporation of the supernatant at 110 °C for 24 h.

All measurements were carried out in duplicate.

#### 2.2.3. Evaluation of Dough Rheology

For the evaluation of rheology, pastes were prepared using the Rapid Visco^®^ Analyser RVA-4C (Newport Scientific Pty. Ltd., Warriewood, Australia), with 12 g sample and 25 g distilled water, at 30 °C. The paddle speed was 960 rpm for the first 10 s of the analysis and then kept at 160 rpm throughout the process, which lasted 3 min. This process was performed in duplicate.

For the analyses of dough rheology under heating conditions, pastes were also prepared with the RVA. First, 5 g of sample was added to 25 mL of water. The mixture was heated to 50 °C for 1 min and then to 95 °C, with a rate of temperature increase of 12 °C/min. Once 95 °C was reached, the temperature was maintained for 2.5 min, after which the suspension cooled down to 30 °C at the end of the analysis. The rotational speed was 960 rpm during the first 10 s of the analysis and was subsequently maintained at 160 rpm throughout the process. This process was performed in duplicate.

Rheological measurements were performed with a Haake RheoStress 1 controlled-stress rheometer (Thermo Fischer Scientific, Scheverte, Germany), equipped with a Phoenix II P1-C25P temperature control unit. Here, 80-mm diameter parallel titanium plates with rough surface geometry were used in order to avoid the displacement of the sample. Vaseline was applied during the analysis (Panreac Química S.A., Castellar del Vallés, Spain) to prevent the sample from drying. After allowing the sample to stabilise for a period of 500 s, a strain scan (0.1–100 Pa) with a constant frequency, 1 Hz, was performed to determine its linear viscoelastic region. For the characterisation of the samples, the following were calculated: G’ or elastic modulus (Pa), G’’ or viscous modulus (Pa) and tangent δ (G’/G’’).

The measurements were carried out in duplicate.

#### 2.2.4. Gel Properties

Texture of the gels was measured with the TA-XT2 texture analyser (Stable Microsystems, Surrey, UK), equipped with the “Texture Expert” software. A penetration test was performed, in which a P/6 probe (SMS Genuine Parts) penetrated 5 mm into the gel, with a test speed of 0.5 mm/s (1 mm/s as pre- and post-test speed) and a penetration distance of 40 mm, in order to determine the gel strength. Gel colour was measured as flour colour. The measurements were carried out in duplicate.

#### 2.2.5. Statistical Analysis 

Data were subjected to a multivariate analysis of variance (MANOVA) using the Fisher LSD (Least significant difference) test (*p* < 0.05) with Statgraphics Centurion XVII software (StatPoint Technologies, Warrenton, USA). Two factors were considered: the type of bread and the area of the bread (crumbs or crust) used to obtain the flours. Significant interactions between both factors were found in none of the parameters involved. Pearson correlations were also carried out to identify relationships among the factors studied.

## 3. Results and Discussion

### 3.1. Particle Size, Microstructure and Colour of the Flours

Table 2 shows the values of the physical properties of the flours obtained, as well as their comparison with a common wheat flour. In terms of particle size, it should be noted that all flours obtained from stale bread (SB) have higher values than those of wheat flour. This may be due to the milling method. Wheat flours are obtained by a progressive milling system to separate the outer layers of the grain completely, and the final separation sieve is about 200 microns. SBs were obtained by a hammer mill, with a 1000-micron sieve. If the milling process were different, or the sieves different, finer flours could be obtained from the SB. However, it must be taken into account that SBs have a certain hardness due to the formation of supramolecular structures related to polymer crystallisation processes associated with amylopectin retrogradation [24]. There are no significant differences between flours obtained from crumbs and flour from crusts, while differences between the flours of the various breads are small. Only the smaller particle size of the flours obtained from pan loaf stands out, although there are no significant differences from those of ciabatta, loaves with additives or small breads. This may indicate that ingredients in pan loaf, such as fat, may soften the product and favour a finer particle size at milling. Particle size has been shown to be a very important factor when making products such as biscuits, both with wheat flour [19] and with gluten-free flour [25], or cakes [26]. The fact that no major differences are observed makes it easier to mix these flours and obtain a flour that is homogeneous in terms of this parameter, with the exception of pan loaf flours, which would be somewhat finer.

**Figure 1:**
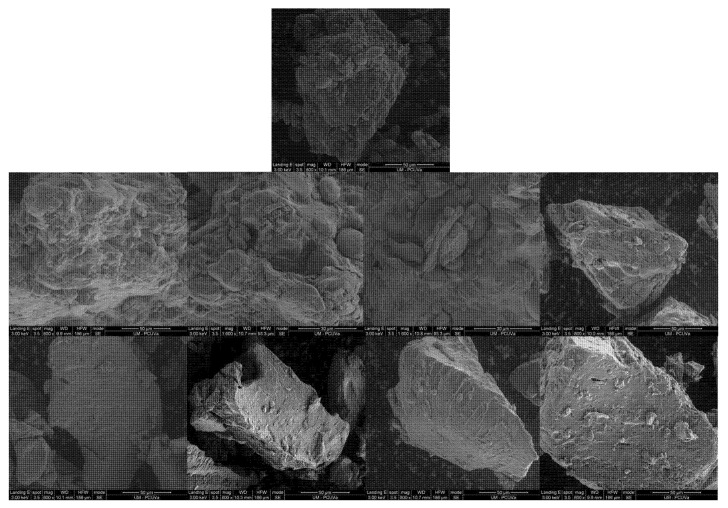
Microstructure of crumb and crust flours. In the upper part, regular flour; in the middle row, crust flours; and in the lower row, crumb flours. From right to left, loaf of bread with additives, “candeal” white bread, wholemeal loaf and pan loaf.

Figure 1 presents the differences between the microstructure of some of the flours obtained. In general, it is observed that crust flours have a higher number of visible starch granules than flours obtained from crumbs, where the level of humidity is higher and, thus, starch has gelatinised and broken due to the higher humidity present in the crumbs [27]. Although these differences have already been observed by Martínez et al. (2018) [13], in our study, we found a lower number of starch granules in the crust flours than in their work. This may be due to the fact that in industrial processes, it is common to incorporate steam at the beginning of baking [28], which could facilitate further gelatinisation of the starch in the outer layers. In fact, Martínez et al. (2018) [13] observed lower starch gelatinisation in the crusts of breads with less hydration. A lower number of starch granules is observed in the crusts analysed in the case of pan loaf, probably because when baking inside a mould, there is a greater retention of humidity, which would facilitate the gelatinisation of the starch. Nevertheless, it should be noted that all SB flours showed complete gelatinisation of the starch under analysis with DSC (Differential scanning calorimetry) (data not shown). This indicates that although some starch granules are visible and, therefore, have not been completely broken, all of them have been gelatinised. Among the crumbs, only the breads obtained from wholemeal flours can be highlighted, as wheat bran fibrous structures can be observed. With the milling system employed, this has not resulted in coarser flours. However, if the aim is to obtain finer flours, it could pose a problem because of the greater resistance of these fibrous structures to breakage [29].

In respect to flour colour, those coming from the crusts are darker (lower L* value) and browner (higher a* and b* values). The higher temperatures reached in the external part, which facilitate the development of the Maillard and sugar caramelisation reactions, explain these differences [30]. Regarding bread varieties, the greatest difference was observed in the flours obtained from wholemeal loaves, which are darker and have higher a* and b* values. This is expected due to the darker colour of the wheat bran as compared to the endosperm and, therefore, of wholemeal flours as compared to refined or white flours, although the colour of the bran and, therefore, of the flours and their by-products may depend on the variety of wheat used [31]. The differences between the other flours are small and only the clearer value of the flours obtained from small breads stands out. These variations may be due to different baking conditions, as higher temperatures or longer baking times result in darker products [32]. In fact, the small breads had a lighter crust, which may indicate a shorter bake.

### 3.2. Hydration Properties

Hydration properties are quality parameters of flours related to their suitability for the production of goods such as biscuits, as they are directly related to the rheology of the doughs and the expansion of these in the baking process [19,33].

As shown in Table 3, the cold hydration properties of flours obtained from SBs are considerably greater than those of a traditional flour. The gelatinisation of starch produced in the baking process accounts for these differences [13]. In fact, flours obtained by processes that favour the gelatinisation of starch have higher hydration values than untreated flours [18,34]. However, compared to the flours obtained by Martínez et al. (2014) [18] with a higher degree of gelatinisation, those obtained from SB show somewhat lower values. This may indicate overtreatment, as starch granules in the gelatinisation process, after swelling, undergo a process of breakage and decrease their thickening power. The treatments to obtain pre-gelatinised flours usually give priority to maintaining the thickening capacity of the flours in the cold. However, the duration of the baking process is usually more than 20–30 min, and although the starch does not reach the temperature of gelatinisation inside the piece until after a few minutes, overheating may take place once the starch has gelatinised. No significant differences have been found between the various flours of SB crusts and crumbs. A slight difference could be expected as, although gelatinisation and, therefore, the swelling of starch granules occur in the crust, the evaporation of water and the lack of water on the outside of the breads make gelatinisation processes shorter, as mentioned. This can be confirmed by the presence of some unbroken granules as seen in the microstructure. However, these subtle differences are not sufficient to affect the cold hydration properties significantly. Another possible explanation is that the changes caused by Maillard reactions may slightly increase the water absorption capacity, depending on the components involved [35], and may compensate for the effects of reduced breakdown of the starch granules in the crust.

Although significant differences were observed between the various types of breads, more so in the WHC or SV than in the WBC, these are scarce. The higher WHC values of the small breads and, especially, the lower values of the pan loaf are noteworthy. The higher values of the small breads may be related to the shorter baking times they require as a result of the shorter distance between the external area and the centre and the possible reduction in starch degradation phenomena and subsequent changes in cooling [36]. Nevertheless, this has not resulted in higher water absorption values after centrifugation (WBC). In the case of the pan loaf, the presence of ingredients such as oil can reduce the capacity to absorb water, which is more closely linked to starch and protein. However, the greater degradation of starch observed in the microstructure of the crust may also have an influence due to the greater retention of humidity in the baking process when performed inside a pan. SB flours obtained from pan loaves also present lower WBC values. Flours obtained from wholemeal loaves exhibit some of the lowest WHC values, with no significant differences from other flours such as the ones obtained from rustic bread or ciabatta. Furthermore, with regard to SV and WBC, no significant differences are observed in comparison to most of the breads analysed. This may be the result of bran being able to increase these values compared to untreated flours, and therefore, wholemeal flours have a greater capacity to absorb water [37]. Gelified starch has the same effect [18], and these flours have a lower proportion of starch. Both effects can therefore be compensated for.

As for WAI, water absorption after heating, no difference was observed between SB and wheat flours. This may be due to the fact that during heating with water, starch gelatinisation in the flour increases the water absorption capacity, in the same way that starch gelatinisation during the baking process does for SB. In contrast to hydration properties in cold conditions, in the WAI, significant differences were observed between crumb and crust flours, with crumb flours being superior. These slight changes may be related to the modification of the small fractions of starch that remained ungelled and can be observed in the microstructure, as mentioned. With regard to the different types of bread, the lower values of WAI in pan and wholemeal loaf flours, both with a lower starch content resulting from the addition of bran or other ingredients, can be highlighted. This confirms the role of gelled starch in hydration properties. Ciabatta flour has the highest WAI value, followed by the classic loaf, but these differences do not exceed 0.55 in most breads, except for wholemeal and pan loaves.

### 3.3. Rheology of Doughs and Pastes

#### 3.3.1. Properties under Cold Conditions

The rheology of doughs made from SB flours is shown in Table 4. In this scenario, the doughs made with common flour were excessively liquid and it was not possible to analyse their rheology with the equipment used in the rheometer. No significant differences were observed between viscosity values, G’ and G” (cold) of crumbs and crusts, although the latter presented higher values of Tg δ. Among the various breads, the pan loaf was the one with the lowest viscosity values, G’ and G” and a higher Tg δ. For the Tg δ, these differences are significant compared to the rest of the breads, which present little differences between them. In the case of G’ and G’’, there are significant differences between the pan loaf flours and the rest, except for rustic bread and wholemeal loaf. Among the other breads, there are no significant differences in G”, and those observed among the values of G’ are much smaller. In general, when excluding pan loaf, wholemeal loaf and rustic bread, the differences between the various SBs are much lower. This can facilitate mixing to obtain homogeneous flours.

The rheological values of the doughs are closely related to the water absorption capacity of the flours. In fact, pan loaf flour, with the lowest values of viscosity, G’ and G”, was also the one that gave the lowest values of WHC, SV and WBC, while the classic loaf flour (highest values of viscosity, G’ and G”) was the one that obtained the highest values of WBC and SV, and WHC was the second highest. In fact, significant correlations (99% at least) have been found between WHC and WBC values and G’, G” and Tg δ, with r values higher than 0.70.

In the literature, various articles explain the possible differences between the rheology of doughs in term of the effect of certain ingredients, but it must be taken into account that these changes are related to the formation of the gluten network and the effect of these ingredients on this network. However, in the case of SB flours, gluten is denatured to obliterate the capacity to form a viscoelastic network. Therefore, the rheology of these doughs is more related to their composition and the thickening power of the different components, especially starch. Thus, the differences observed between the doughs may be due to the presence of components that reduce this thickening capacity, such as oil in the case of pan loaf or bran in the case of wholemeal loaf. However, these differences can also be caused by the degradation of gelatinised starch by excessive treatment, as discussed in the case of hydration properties, or by the enzymatic hydrolysis of starch [38], either by the action of a greater number of amylases of the flour itself or added with improvers, or by longer processes that have allowed greater degradation. The use of sourdoughs can also contribute to the degradation of starch, not only because of the increased enzymatic action but also because of its higher acidity and the possible acid hydrolysis of starch [39], as well as the presence of other acids. In fact, the classic loaf and the small breads, which have the highest values of G’ and G”, do not contain enzymes among their ingredients, nor other additives where acids can be included, nor sourdough. However, they do contain malted flours, which may have amylase activity.

#### 3.3.2. Properties under Heating Conditions

As can be seen in Table 4, in doughs made from SB flours, the values of G’ and G” are higher and values of Tg δ are lower than those made from wheat flour. On the other hand, crust flours present higher values of G’ and lower values of Tg δ than crumb flours but no significant differences in G”. Among the several bread types, pan and wholemeal loaves have lower G’ and G” values, followed by the loaf with additives and the pan loaf. Small breads and “candeal” bread stand out because of their high values.

As with cold rheology, the G’ and G” values of doughs after heating correlate with hydration values, but, in this case, with those obtained after heating (WAI). In the case of G”, the correlation is significant at 99% (r = 0.62), while for G’, at 95% (r = 0.58), and therefore, the correlations are somewhat weaker than those found for cold properties. The rheological properties under heat are influenced by starch gelatinisation processes, which can explain the differences between crust and crumb since, unlike crumbs, there are a small number of ungelled starch granules in the SB crust flours. However, starch retrogradation reactions, especially amylose, also have an influence. In this case, the presence of other ingredients in the bread and the decrease in the amount of starch, as in wholemeal breads and pan loaves, could explain the lower values of these breads. On the other hand, the presence of bran particles may interfere with the creation of a more compact and ordered structure, which would explain the lower values of these flours. The differences between the other bread types, as has already been mentioned for other factors, may depend on starch degradation phenomena during the baking process, which will depend on the amylase activity, pH of the doughs and fermentation and baking times. Thus, the flours with the highest values are those of “candeal” bread, made with low hydration (less than 50 parts of water for every 100 parts of flour) and small breads (with shorter baking times). Therefore, it seems that these factors can minimise the degradation of starch, although further studies would be needed to confirm this. Except for these breads, differences between the rest of the bread flours and flours from classic bread—the most widely produced and from which the greatest waste is generated—are scarce, although in some cases, they are significant.

### 3.4. Gel Properties

Table 5 shows the properties of the gels obtained from SB flours. In terms of colour, the gels obtained from crust flour are darker than those obtained from crumb flour and have higher a* and b* values. This corresponds to the darker colours and higher a* and b* values of the crust flours in comparison to the crumbs. Similarly, among the different bread types, those that gave rise to darker flours (wholemeal and, to a lesser extent, rustic bread) produce darker gels and those that generated lighter flours (small breads) create lighter gels. The colour of the gels is not the same as that of the flours, due in part to dilution with water, and in part to changes in gelatinisation, which in the case of bread flours is much lower, since the starch has gelatinised during baking. It seems that this higher gelatinisation of wheat flour reduces the L value (which is why it is lower than that of crumbs) and can be compensated by the higher L values of crust flours. Therefore, the colour of the gels is directly related to the colour of the flours from which they are obtained, and their differences are mainly due to Maillard reactions, as mentioned above. In the process of gel making, the temperatures do not exceed 100 °C and there are no Maillard or caramelisation reactions that could modify the colour. The absence of the Maillard reaction can be also explained by the moist conditions within the crumbs, which reduces the combinations between sugars and amino acids. Thus, the L*, a* and b* values of flours and gels have a significant correlation at 99.9%, with r=0.83, 0.98 and 0.93, respectively.

Regarding the hardness of the gels, those obtained from SB flours present lower values than those obtained from wheat flour. This is reasonable since the ability to form gels in wheat flours relies on the starch, and the baking process partially degrades this starch by reducing its ability to gel. Gels obtained from crust flour are somewhat harder than those obtained from crumb flour. This corresponds to the higher water absorption values (WAI) of crust flours under heating, as opposed to crumb flours. The reason for these higher values is that the small percentage of starch granules that have not degraded or have degraded to a lesser extent in the baking process within the crust increase the hardness of the gels when heated.

Among the different breads, the hardest gels were obtained with “candeal” bread and small breads, while with the wholemeal and pan loaves flours, hardly any gels were obtained, only a viscous dough with a low resistance to deformation or a very weak gel. In the production of gels, firmness increases with time due to the changes that amylose undergoes after gelatinisation, from a more amorphous structure to a more ordered and crystalline state [40]. However, the presence of other components, besides reducing the total starch content, can also interfere with amylose retrograde reactions [40,41], which would explain the low hardness values of pan or wholemeal loaves.

The previous degradation of starch, such as that produced by acidification [42], also reduces the hardness of the gels obtained. This would explain the lower hardness of SB gels compared to wheat flour gels and the differences between the flours of some breads due to different processing conditions, which may include different dough pH values and fermentation times.

As mentioned, the hardness of the gels obtained is related to the gelatinisation reactions of the starch and to its amount in the sample. In fact, significant correlations have been found between this parameter and most of the hydration and rheology parameters studied, also related to starch and its modifications. Among the hydration properties, the correlation between the hardness of the gels and both WHC (r = 0.76) and WAI (r = 0.73) stands out. Regarding the rheological properties, as would be expected, the highest correlations are found between the hardness of the gels and the rheology of the pastes after heating. Specifically, there are significant correlations at 99.9% between G’ (r = 0.86) and G” (r = 0.92) and the hardness of the gels obtained.

## 4. Conclusions

Flours obtained from stale bread have shown a greater capacity to absorb water under cold conditions and the G’ and G” values of their doughs are higher than those of wheat flours, but they possess less gelling power. In this work, it is also shown, for the first time, that the characteristics of different commercial breads in terms of hydration properties, rheology of the pastes or doughs obtained in cold or hot conditions and gel properties are quite similar. Therefore, different breads produced in the same facilities could be mixed to obtain flours with homogeneous characteristics. However, those breads containing ingredients such as fats, oils or bran should be excluded, and other factors which, in specific cases, may modify the characteristics of the flours should be taken into account, such as previous enzymatic, heat or acidic degradation. It has also been proven that despite the differences of crumb and crust flours both in colour—due to the Maillard and caramelisation reactions developed in the baking process—and microstructure, these differences do not translate into substantial ones in the rheological properties of the flours obtained. Finally, it has been observed that in these cases, a simple analysis such as that of the WHC or WBC (WAI for hot properties) can predict, or give an idea of, the rheological properties of the flours, which are usually measured with more complex and expensive equipment. This would facilitate the quality control of these flours. These results can be very useful for reusing breads that have gone stale or been wasted in distribution, thus reintroducing them into the human food chain to produce biscuits, batters or other products.

## Figures and Tables

**Table 1:** Ingredients of the breads analysed.

	Wholemeal Loaf	Classic Loaf	Loaf with Additives	Ciabatta	Small Breads	“Candeal” Bread	Pan Loaf	Rustic Bread
Sunflower oil							X	
Sugar							X	
Emulsifiers	X		X		X	X	X	
Enzymes	X		X			X		X
Plant extracts	X							
Wheat gluten	X							
Wheat flour	X	X	X	X	X	X	X	X
Malted flour		X		X				
Baker’s yeast	X	X		X	X	X	X	X
Sourdough			X	X				
Other additives *	X		X	X		X	X	X
Salt	X	X	X	X	X	X	X	X
Seeds	X							

* Antioxidants, preservatives, acidity regulators, flour treatment agents and anti-caking agents.

**Table 2:** Particle size and colour of the flours obtained.

	D [4,3]	L*	a*	b*
Flour	97.20 ± 0.00	89.75 ± 6.39	1.62 ± 0.25	10.80 ± 0.35
Crumb	206.19 a	84.48 b	3.20 a	15.13 a
Crust	193.38 a	81.15 a	7.73 b	21.40 b
*Error*	7.39	0.43	0.24	0.40
Wholemeal loaf	233.75 b	71.05 a	8.95 c	20.97 d
Classic loaf	217.50 b	85.44 cd	4.57 ab	17.86 abc
Loaf with additives	191.50 ab	83.87 bc	5.75 b	18.80 bcd
Ciabatta	197.75 ab	84.25 bcd	5.22 b	18.81 bcd
Small breads	192.25 ab	86.62 d	3.58 a	16.49 ab
“Candeal” bread	201.00 b	84.30 bcd	5.22 b	19.75 cd
Pan loaf	155.75 a	84.33 bcd	5.07 b	15.87 a
Rustic bread	208.75 b	82.68 b	5.32 b	17.55 abc
Error	14.78	0.86	0.47	0.79

Within each column and for each factor, values with same letter do not differ significantly (*p* < 0.05).

**Table 3:** Hydration properties of the flours obtained.

	WHC	SV	WBC	WAI
Flour	1.39 ± 0.21	2.23 ± 0.02	0.92 ± 0.01	4.57 ± 0.01
Crumb	3.15 a	3.84 a	1.95 a	4.39 a
Crust	3.18 a	3.84 a	2.05 a	4.69 b
Error	0.05	0.06	0.05	0.05
Wholemeal loaf	2.95 b	3.83 bc	2.02 b	4.08 a
Classic loaf	3.51 de	4.11 cd	2.26 b	4.80 cd
Loaf with additives	3.24 bcd	3.96 bcd	2.05 b	4.49 b
Ciabatta	3.00 b	3.71 b	1.99 b	5.04 d
Small breads	3.66 e	4.29 d	2.03 b	4.65 bc
“Candeal” bread	3.31 cd	3.92 bc	2.00 b	4.67 bc
Pan loaf	2.53 a	3.20 a	1.60 a	4.00 a
Rustic bread	3.10 bc	3.68 b	2.05 b	4.61 bc
Error	0.10	0.11	0.10	0.10

WHC (Water-Holding Capacity). SV (Swelling Volume). WBC (Water-Binding Capacity). WAI (Water Absorption Index). Within each column and for each factor, values with same letter do not differ significantly (*p* < 0.05).

**Table 4:** Rheology and viscosity of pastes.

	Cold			Heating		
	G’	G’’	tan δ	G’	G’’	tan δ
Flour				398 ± 107	70 ± 5	0.18 ± 0.04
Crumb	1911.57 a	314.41 a	0.18 a	808.91 a	123.48 a	0.16 b
Crust	1916.69 a	300.30 a	0.21 b	1188.01 b	157.20 a	0.14 a
Error	337.26	46.60	0.01	36.59	4.16	0.001
Wholemeal loaf	1025.86 ab	171.26 ab	0.19 c	319.88 a	50.63 a	0.16 cd
Classic loaf	3966 c	567.58 c	0.15 a	1053.33 d	148.23 c	0.14 b
Loaf with additives	2073.84 b	341.69 bc	0.17 abc	697.9 b	107.85 b	0.16 cd
Ciabatta	2665.15 bc	423.98 bc	0.17 abc	991.75 cd	147.35 c	0.15 bc
Small breads	2600.13 bc	406.35 bc	0.16 ab	1367.25 e	179.95 d	0.13 a
“Candeal” bread	1545.87 ab	304.95 abc	0.20 c	1915.5 f	247.75 e	0.13 a
Pan loaf	197 a	45.71 a	0.35 d	773.3 bc	102.81 b	0.13 a
Rustic bread	1239.21 ab	197.37 ab	0.19 bc	868.8 bcd	138.18 c	0.16 d
Error	674.51	93.21	0.01	73.17	8.33	0.003

G’ (elastic modulus); G’’ (viscous modulus); tan δ (G’’/G’). Within each column and for each factor, values with same letter do not differ significantly (*p* < 0.05).

**Table 5:** Gel properties.

	L*	a*	b*	Hardness (N)
Flour	61.15	−0.95	4.74	0.39
Crumb	67.14 b	−0.14 a	5.86 a	0.20 a
Crust	61.14 a	4.29 b	13.76 b	0.25 b
Error	0.51	0.23	0.39	0.01
Wholemeal loaf	54.51 a	6.89 c	14.93 d	0.03 a
Classic loaf	65.10 bc	0.81 ab	8.09 ab	0.29 e
Loaf with additives	63.74 bc	1.98 b	9.91 bc	0.20 c
Ciabatta	65.98 bcd	1.22 ab	9.19 abc	0.23 cd
Small breads	68.64 d	0.07 a	7.41 a	0.34 f
“Candeal” bread	65.93 bcd	2.02 b	10.95 c	0.38 g
Pan loaf	66.72 cd	2.16 b	9.57 abc	0.07 b
Rustic bread	63.28 b	1.42 ab	8.42 ab	0.24 d
Error	1.03	0.46	0.77	0.01

Within each column and for each factor, values with same letter do not differ significantly (*p* < 0.05).

## Data Availability

The data presented in this study are available on request from the corresponding author.
